# Editorial

**Published:** 1914-07

**Authors:** 


### EDITORIAL

The Business Office of the *Journal-Record* is 23 West Alabama Street.

The Editorial Office is 1014-15 Atlanta National Bank Building.

Address all Business Communications to *Journal-Record of Medicine,* 23 West Alabama Street.

Make remittances payable to *The Journal-Record of Medicine.*

On matters pertaining to the Editorial and Original Communications, address **Edgar G.** Ballenger, M. D., Atlanta, Ga.

Reprints of Original Articles will be furnished at cost price. **Requests for the same** should always be made in THE MANUSCRIPT.

We will present, postpaid, on request to each contributor of an original article, twenty (20) marked copies of *The Journal-Record of Medicine* containing such article.

### PUBLIC HEALTH IN GEORGIA.

The House has passed the Ellis bill which provides for a State Board of Health and for District Commissioners in the various Counties.

It is to be commended for this and we hope the Bill will become a law with little change in the Senate. It might be well to modify the method of appointment of District Commissioners so that it would be mandatory upon each district to have them forthwith instead of waiting until two successive Grand Juries were convinced that the health of every locality needed care and attention. We can not see why a Grand Jury should have a thing to do with such a procedure and we do know that whenever such questions have arisen in different parts of the State, it has been men who did not and who could not understand questions of sanitation and hygiene—men who are likely to be upon Grand Juries, who have stood in the way just because they did not know any better. The spread of malaria in Georgia to communities who were warned against it by competent authorities has proved this contention time and again.

However, the idea that some one or something must be indicted for the deplorable conditions of public health in various parts of the State is a good one and perhaps that was in the mind of the framers of the Bill quite as much as a subserviency to the old method of doing things by jury instead of just getting them done.

Large powers are given to the Board and Commissioners and it remains with the thoughtful citizens to see to it that these powers are exercised with dispatch as well as discretion in their neighborhoods. The Profession has a duty in so leading such men that there will be a strong sentiment to uphold the State in its health work. People should be made to understand that nowhere is the concern of one so much the concern of all as it is in the prevention of disease.

The public lectures required by the Bill should be supplemented extensively by progressive members of the Profession throughout the State and citizens should know how disease spreads and what it means for one of their number to have tuberculosis or malaria or meningitis or a simple "cold." They should be taught that the reporting and isolating of contagious diseases should not be judged by the immediate inconvenience to the sufferer and his family, but by the effect upon everyone. They should be shown that in the matters of dollars and cents, it would be far more economic to pay a man's expenses and those of his family when he was sick of some infectious disease than it would be to let him run free just because he was poor, and thus infect several others. All this is plain to the thoughtful physician, but often enough it doesn't seem good policy for some reason or another, for him to make it plain to others. Thus the question has demanded an answer these many years throughout Georgia.

To accomplish real good, a bureau of Vital Statistics should be established in the State and under the complete authority of the act, every contagious disease should be reported together with all births, deaths, marriages and such other facts as are needed to let us all know how we are getting on. Then we can know where we are. We can see at once where epidemics are starting and make instant efforts towards stopping them. In New Orleans at the present moment, they are stopping the Bubonic Plague. Why? Because they recognized the fact it had come to them and announced methods of protection that everyone could avail himself of.

Let us, therefore take stock and do it all the time so that we can make a business of keeping well and be successful at it. It will cost some money but we will know where that goes to. Disease would cost and does cost thousands and millions more and no one knows what becomes of it.

We hope the Bill will become a law and that the people will so stir their Grand Juries that those bodies will see that health is even more important for their consideration than crime. Then they will know that health prevents crime just as it yields happiness.

